# Comparative efficacy of Bacillus Calmette–Guérin instillation and radical cystectomy treatments for high-risk non-muscle-invasive urothelial cancer classified as high-grade T1 in initial and repeat transurethral resection of bladder tumor

**DOI:** 10.3389/fonc.2024.1394451

**Published:** 2024-06-18

**Authors:** Song Zhen, Chen Hao, Yu Yanhang, Lin Yuxin, Ouyang Jun, Zhang Zhiyu

**Affiliations:** ^1^ Department of Urology, The First Affiliated Hospital of Soochow University, Suzhou, China; ^2^ Department of Urology, Taixing People’s Hospital, Taizhou, China

**Keywords:** high-risk non-muscle-invasive urothelial cancer, Bacillus Calmette-Guérin instillation, radical cystectomy, overall survival, progression-free survival

## Abstract

**Objective:**

To compare the differential therapeutic effects of Bacillus Calmette-Guérin (BCG) instillation and radical cystectomy (RC) for high-risk non-muscle–invasive urothelial cancer (NMIBC) classified as high-grade T1 in initial and repeat transurethral resection of bladder tumors (TURBT) and to construct a prediction model.

**Methods:**

We retrospectively analyzed the clinical data of patients with malignant bladder tumors treated at the First Affiliated Hospital of Soochow University from January 2016 to December 2017 and compared the differences in 1-year, 2-year, 3-year, 5-year, and comprehensive overall survival (OS) and progression-free survival (PFS) between BCG instillation treatment and RC treatment. Survival curves were drawn to show differences in OS and PFS between the two groups. Concurrently, univariate and multivariate COX analyses were performed to identify risk factors affecting OS and PFS, and a nomogram was created.

**Results:**

In total, 146 patients were included in the study, of whom 97 and 49 were in the BCG and RC groups, respectively. No statistical differences were observed in the 1- and 2-year OS and PFS between the two groups, whereas significant statistical differences were found in the 3-year, 5-year, and comprehensive OS and PFS. Survival curves also confirmed the statistical differences in OS and PFS between the BCG and RC groups. Multivariate COX analysis revealed that the treatment method, concomitant satellite lesions, and albumin-to-alkaline phosphatase ratio (AAPR) were independent risk factors affecting OS and PFS. The nomogram that was further plotted showed good predictive ability for OS and PFS.

**Conclusion:**

For patients who exhibit high-level T1 pathology after both initial and repeat TURBT, especially those with low AAPR, and concomitant satellite lesions, choosing RC as a treatment method offers a better prognosis.

## Introduction

Bladder cancer, the ninth most prevalent cancer worldwide, has an alarming estimated 430,000 new cases annually (1). For patients initially treated for bladder tumors, if the pathology results from the first transurethral resection of the bladder tumor (TURBT) indicate a high-grade T1 stage tumor, then conducting repeat TURBT (re-TURBT) can significantly increase progression-free survival (PFS) and disease-free survival rates (2, 3). However, for patients whose pathology results from re-TURBT are still in the high-grade T1 stage, the recommended course of action is either Bacillus Calmette-Guérin (BCG) instillation therapy or radical cystectomy (RC) (4). However, the superiority of either of these treatment options remains controversial (5–7).

Nutritional indicators, including the prognostic nutritional index (PNI) and the controlling nutritional status (CONUT) score, have recently been found to be associated with the prognosis of bladder tumors (8, 9). At the same time, inflammatory factors and their related derivative indicators, including albumin-to-alkaline phosphatase ratio (AAPR), neutrophil-lymphocyte ratio (NLR), platelet-lymphocyte ratio (PLR), lymphocyte-monocyte ratio (LMR), and systemic immune-inflammation index (SII) have also recently been found to be associated with the prognosis of bladder tumors (10–12). Our study further validated the relationship between these indicators and the PFS and overall survival (OS) after bladder cancer surgery.

Our study mainly aimed to follow up patients with high-risk non-muscle-invasive urothelial cancer (NMIBC) who were diagnosed with high-level T1 stage via two TURBT pathology results. After receiving either BCG instillation or RC therapy, we compared the differences in OS and PFS between the two groups of patients. This study aimed to provide a useful reference for future clinical decision-making for this type of patients.

## Materials and methods

### Clinical data

This was a single-center, retrospective study. We collected and analyzed data from patients who underwent TURBT at the First Affiliated Hospital of Soochow University between January 2016 and December 2018. The inclusion criteria were as follows: (1) having undergone both initial TURBT and re-TURBT, with both pathologies being high-grade T1 stage tumors; (2) not accompanied by carcinoma *in situ*; and (3) not including all three clinically relevant risk factors; age >70 years, multiple papillary tumors, and diameter >3 cm. The exclusion criteria were as follows: (1) immunodeficiency or active tuberculosis; (2) allergy to BCG; and (3) refusal to participate in the study. In total, 146 patients were included in this study. Among them, 97 received BCG instillation, and 49 underwent RC treatment. We followed up with all enrolled patients for a period ranging from 10 to 84 months, with an average follow-up duration of 54.21 ± 20.29 months.

The clinical data collected includes sex, age, body mass index (BMI), high blood pressure (HBP), diabetes mellitus (DM), hyperlipidemia, smoking history, tumor multiplicity, tumor diameter, concurrent satellite lesions, PNI, CONUT score, AAPR, NLR, PLR, LMR, and SII. The calculation formula for PNI is PNI= Serum Albumin (g/L) + 5 x Total Peripheral Blood Lymphocyte Count (x10^9^/L). The CONUT score is calculated based on serum albumin levels, total cholesterol levels, and total lymphocyte count, with a total score ranging from 0–12 points. The calculation formula for AAPR is the pre-operative ratio of albumin to alkaline phosphatase. The calculation formula for SII is SII = Platelet Count x Neutrophil Count/Lymphocyte Count in the blood.

This study adhered to the ethical standards stipulated in the Declaration of Helsinki, which was revised in 2013. Ethical approval was obtained from the Ethics Committee of the First Affiliated Hospital of Soochow University (Approval ID: No. 113[2024]). Before the study, all participating patients signed informed consent forms.

### Treatment protocols

RC Group: After the initial TURBT and re-TURBT, each patient underwent preoperative chest, abdominal, and pelvic computed tomography (CT) scans to ensure the absence of distant metastases. Patients were deemed eligible if their Eastern Cooperative Oncology Group (ECOG) performance status was 0–2 points. Patients and their families provided informed consent for surgery, after which RC and urinary diversion were performed under general anesthesia. Details of this procedure have been described previously (13).

BCG Group: After undergoing two rounds of TURBT, all patients underwent chest, abdominal, and pelvic CT scans to rule out distant metastases, with an ECOG score ranging from 0 to 2. After the patients and their families were fully informed and signed consent forms were obtained, the BCG instillation therapy was initiated. The instillation began two weeks after re-TURBT and was conducted in the following sequence: (1) induction instillation, once a week, for a total of six times with 120 mg each instillation; (2) intensification instillation, once every two weeks for three sessions; maintenance instillation was performed once a month for a total of 10 sessions; and (3) continuous instillations were performed once a month for three continuous years.

### Follow-up

All patients underwent examinations every three months for the first two years, semi-annually from the second to the fifth year, and annually thereafter. These examinations included chest, abdominal, and pelvic CT and urinary cytology tests. In addition, patients who received BCG instillation therapy underwent supplementary cystoscopy. None of the participants withdrew midway through the study period. For the RC group, the occurrence of tumor recurrence or progression during the follow-up period is considered as the endpoint event for PFS. For the BCG group, the occurrence of high-grade recurrence or tumor progression during the follow-up period is considered as the endpoint event for PFS. And death was regarded as the endpoint event for OS for both two groups.

### Statistical analysis

R software (version 4.2.1) with related accessory packages including “ggplot2[3.3.6], survival[3.3.1], rms[6.3–0], stats[4.2.1], ResourceSelection[0.3–5]” was utilized in this study. Qualitative data were compared between the two groups using a t-test. Cumulative data between the two groups were examined the χ2 test. Kaplan–Meier and log-rank tests were used for survival analysis. Univariate and multivariate COX regression analyses were used to identify independent risk factors. A nomogram model was established to predict the OS and PFS. A calibration curve was constructed to evaluate the model. Statistical significance was set at p < 0.05. The sample size was calculated using R software, which required significant results from 103 participants in our study (14). Two senior statisticians independently verified the data.

## Results

### Baseline levels comparison between the BCG and RC groups

There were no statistically significant differences between the two groups in terms of sex, age, BMI, HBP, DM, hyperlipidemia, smoking history, tumor multiplicity, tumor diameter, concomitant satellite lesions, PNI, CONUT score, AAPR, NLR, PLR, LMR, or SII (**Table 1**). Meanwhile, the postoperative pathology of RC group patients includes 39 cases classified as T1N0M0, 6 cases as T2aN0M0, 1 case as T2aN2M0, 1 case as T2bN1M0, 1 case as T2bN2M0, and 1 case as T3aN2M0.

**Table 1:** Comparisons of the clinicopathological characteristics between the BCG and the RC group.

Characteristics	BCG	RC	t/χ^2^ value	P value
n	97	49		
Gender, n (%)			0.149	0.700
Male	85 (87.629%)	44 (89.796%)		
Female	12 (12.371%)	5 (10.204%)		
Age(year), mean ± sd	70.536 ± 11.518	69.061 ± 12.144	0.717	0.474
BMI(kg/m^2^), mean ± sd	23.561 ± 4.015	22.989 ± 3.059	0.876	0.382
HBP, n (%)			1.247	0.264
Yes	42 (43.299%)	26 (53.061%)		
No	55 (56.701%)	23 (46.939%)		
DM, n (%)			0.763	0.382
Yes	25 (25.773%)	16 (32.653%)		
No	72 (74.227%)	33 (67.347%)		
Hyperlipidemia, n (%)			0.485	0.486
Yes	6 (6.186%)	1 (2.041%)		
No	91 (93.814%)	48 (97.959%)		
Smoking, n (%)			0.780	0.377
Yes	41 (42.268%)	17 (34.694%)		
No	56 (57.732%)	32 (65.306%)		
Tumor multiplicity, n (%)			0.586	0.444
Yes	43 (44.330%)	25 (51.020%)		
No	54 (55.670%)	24 (48.080%)		
Tumor diameter(mm), mean ± sd	31.567 ± 13.898	35.673 ± 12.873	-1.727	0.086
Concomitant satellite lesions, n (%)			0.274	0.600
Yes	48 (49.485%)	22 (44.898%)		
No	49 (50.515%)	27 (55.108%)		
PNI(%), mean ± sd	48.534 ± 5.803	48.814 ± 7.011	-0.257	0.798
CONUT score, mean ± sd	1.6082 ± 1.7051	1.6531 ± 1.9317	-0.143	0.886
AAPR, mean ± sd	0.555 ± 0.156	0.535 ± 0.174	0.712	0.478
NLR, mean ± sd	3.001 ± 1.320	2.928 ± 1.282	0.320	0.749
PLR, mean ± sd	160.300 ± 78.240	146.410 ± 71.448	1.043	0.299
LMR, mean ± sd	3.370 ± 1.731	3.288 ± 1.884	0.261	0.794
SII, mean ± sd	645.590 ± 352.360	594.320 ± 398.570	0.794	0.429

BCG, Bacillus Calmette-Guérin; RC, radical cystectomy; BMI, body mass index; HBP, high blood pressure; DM, diabetes mellitus; PNI, prognostic nutrition index; CONUT, controlling nutritional status; AAPR, albumin-to-alkaline phosphatase ratio; NLR, neutrophil-lymphocyte ratio; PLR, platelet-lymphocyte ratio; LMR, lymphomonocyte ratio; SII, systemic immunoinflammatory index; sd, standard deviation.

### OS and PFS comparison between the BCG and RC groups

**Figure 1:**
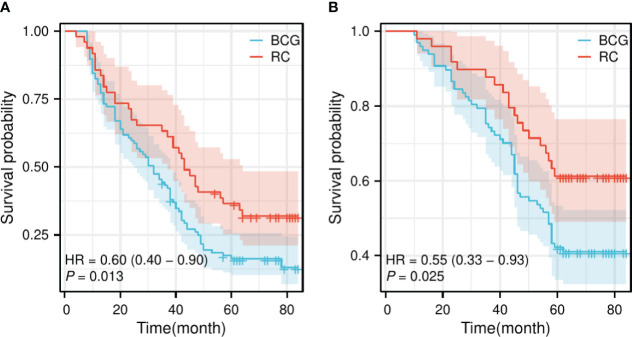
Survival curve of OS and PFS for the BCG and RC groups. **(A)** The survival curve of OS shows a significant difference between the BCG and RC groups. **(B)** The survival curve of PFS also demonstrates a significant difference between the BCG and RC groups. OS, overall survival; PFS, progress-free survival; BCG, Bacillus Calmette-Guérin; RC, radical cystectomy; HR, hazard ratio.

The follow-up period for the BCG group ranged from 10 to 84 months, averaging 51.43 ± 20.50 months, whereas for the RC group, it ranged from 11 to 84 months, averaging 59.71 ± 18.89 months. There was a statistically significant difference in the follow-up time between the two groups (t=2.365, p=0.019). There were no statistically significant differences in the 1-year and 2-year OS (95.876% vs. 97.959% and 83.505% vs. 91.837%, respectively) and PFS (80.412% vs. 85.714% and 58.763% vs. 67.347%, respectively) between the BCG and RC groups. However, statistical differences were found in 3-year, 5-year, and comprehensive OS (75.258% vs. 89.796%, 41.237% vs. 61.224%, and 41.237% vs. 61.224%, respectively) and PFS (43.299% vs. 63.265%, 18.557% vs. 38.776%, and 16.495% vs. 32.653%, respectively) (**Table 2**). The survival curves indicated that the RC group significantly outperformed the BCG group in terms of both OS [Hazard Ratio (HR) = 0.60, p = 0.013] and PFS (HR = 0.55, p = 0.025) (**Figure 1**).

**Table 2:** Comparisons of OS and PFS between the BCG and RC groups.

Characteristics	BCG	RC	χ^2^ value	P value
n	97	49		
1-year OS, n (%)			0.029	0.864
Censored	93 (95.876%)	48 (97.959%)		
Death	4 (4.124%)	1 (2.041%)		
1-year PFS, n (%)			0.625	0.429
Censored	78 (80.412%)	42 (85.714%)		
Recurrence	19 (19.588%)	7 (14.286%)		
2-year OS, n (%)			1.912	0.167
Censored	81 (83.505%)	45 (91.837%)		
Death	16 (16.495%)	4 (8.163%)		
2-year PFS, n (%)			1.015	0.314
Censored	57 (58.763%)	33 (67.347%)		
Recurrence	40 (41.237%)	16 (32.653%)		
3-year OS, n (%)			4.323	0.038
Censored	73 (75.258%)	44 (89.796%)		
Death	24 (24.742%)	5 (10.204%)		
3-year PFS, n (%)			5.191	0.023
Censored	42 (43.299%)	31 (63.265%)		
Recurrence	55 (56.701%)	18 (36.735%)		
5-year OS, n (%)			5.211	0.022
Censored	40 (41.237%)	30 (61.224%)		
Death	57 (58.163%)	19 (38.776%)		
5-year PFS, n (%)			7.034	0.008
Censored	18 (18.557%)	19 (38.776%)		
Recurrence	79 (81.443%)	30 (61.224%)		
Comprehensive OS, n (%)			5.211	0.022
Censored	40 (41.237%)	30 (61.224%)		
Death	57 (58.763%)	19 (38.776%)		
Comprehensive PFS, n (%)			4.967	0.026
0	16 (16.495%)	16 (32.653%)		
1	81 (83.505%)	33 (67.347%)		

BCG, Bacillus Calmette-Guérin; RC, radical cystectomy; OS, overall survival; PFS, progression-free survival.

### Influencing factors of OS and PFS

Univariate COX analysis showed that treatment method, HBP, concomitant satellite lesions, PNI, and AAPR were associated with OS, whereas treatment method, concomitant satellite lesions, PNI, and AAPR were associated with PFS. However, multivariate COX analysis demonstrated that the treatment method, concomitant satellite lesions, and AAPR were independent risk factors for OS and PFS (**Tables 3**, **4**).

**Table 3:** Univariate and multivariate logistic regression analysis of influencing factors on OS.

Characteristics	Total(N)	Univariate analysis		Multivariate analysis	
		Hazard ratio (95% CI)	P value	Hazard ratio (95% CI)	P value
Treatment	146				
BCG	97	Reference		Reference	
RC	49	0.551 (0.328 - 0.927)	0.025	0.553 (0.328 - 0.932)	0.026
Gender	146				
Male	129	Reference			
Female	17	0.966 (0.482 - 1.937)	0.922		
Age(year)	146	0.997 (0.978 - 1.016)	0.762		
BMI(kg/m^2^)	146	1.008 (0.948 - 1.073)	0.789		
HBP	146				
Yes	68	Reference		Reference	
No	78	1.600 (1.007 - 2.543)	0.047	1.549 (0.972 - 2.469)	0.066
DM	146				
Yes	41	Reference			
No	105	0.720 (0.448 - 1.157)	0.174		
Hyperlipidemia	146				
Yes	7	Reference			
No	139	0.810 (0.296 - 2.219)	0.682		
Smoking	146				
Yes	58	Reference			
No	88	0.885 (0.561 - 1.396)	0.600		
Tumor multiplicity	146				
Yes	68	Reference			
No	78	0.782 (0.499 - 1.226)	0.283		
Tumor diameter(mm)	146	1.005 (0.989 - 1.020)	0.568		
Concomitant satellite lesions	146				
Yes	70	Reference		Reference	
No	76	0.514 (0.325 - 0.814)	0.004	0.520 (0.328 - 0.826)	0.006
PNI(%)	146	0.958 (0.924 - 0.994)	0.023	0.966 (0.930 - 1.004)	0.082
CONUT score	146	1.100 (0.980 - 1.235)	0.105		
AAPR	146	0.157 (0.039 - 0.630)	0.009	0.200 (0.046 - 0.872)	0.032
NLR	146	1.063 (0.899 - 1.258)	0.475		
PLR	146	1.000 (0.998 - 1.003)	0.764		
LMR	146	0.979 (0.858 - 1.117)	0.754		
SII	146	1.000 (0.999 - 1.001)	0.850		

BCG, Bacillus Calmette-Guérin; RC, radical cystectomy; BMI, body mass index; HBP, high blood pressure; DM, diabetes mellitus; PNI, prognostic nutrition index; CONUT, controlling nutritional status; AAPR, albumin-to-alkaline phosphatase ratio; NLR, neutrophil-to-lymphocyte ratio; PLR, platelet-to-lymphocyte ratio; LMR, lymphomonocyte ratio; SII, systemic immunoinflammatory index; CI, confidence interval; OS, overall survival.

**Table 4:** Univariate and multivariate logistic regression analysis of influencing factors on PFS.

Characteristics	Total(N)	Univariate analysis		Multivariate analysis	
		Hazard ratio (95% CI)	P value	Hazard ratio (95% CI)	P value
Treatment	146				
BCG	97	Reference		Reference	
RC	49	0.595 (0.396 - 0.895)	0.013	0.568 (0.374 - 0.863)	0.008
Gender	146				
Male	129	Reference		Reference	
Female	17	0.578 (0.301 - 1.107)	0.098	0.659 (0.334 - 1.298)	0.228
Age(year)	146	0.994 (0.978 - 1.009)	0.420		
BMI(kg/m^2^)	146	1.045 (0.993 - 1.100)	0.088	1.032 (0.978 - 1.088)	0.249
HBP	146				
Yes	68	Reference			
No	78	1.120 (0.774 - 1.619)	0.548		
DM	146				
Yes	41	Reference			
No	105	1.052 (0.696 - 1.590)	0.811		
Hyperlipidemia	146				
Yes	7	Reference			
No	139	0.903 (0.396 - 2.059)	0.809		
Smoking	146				
Yes	58	Reference			
No	88	0.926 (0.637 - 1.345)	0.686		
Tumor multiplicity	146				
Yes	68	Reference			
No	78	0.780 (0.540 - 1.127)	0.186		
Tumor diameter(mm)	146	1.000 (0.987 - 1.013)	0.965		
Concomitant satellite lesions	146				
Yes	70	Reference		Reference	
No	76	0.606 (0.418 - 0.878)	0.008	0.628 (0.421 - 0.939)	0.023
PNI(%)	146	0.963 (0.934 - 0.993)	0.015	0.992 (0.940 - 1.047)	0.768
CONUT score	146	1.098 (0.997 - 1.208)	0.058	1.010 (0.835 - 1.223)	0.915
AAPR	146	0.175 (0.055 - 0.554)	0.003	0.259 (0.071 - 0.940)	0.040
NLR	146	1.116 (0.981 - 1.271)	0.096	1.103 (0.918 - 1.325)	0.297
PLR	146	1.002 (1.000 - 1.004)	0.067	1.000 (0.997 - 1.003)	0.990
LMR	146	0.921 (0.822 - 1.033)	0.159		
SII	146	1.000 (1.000 - 1.001)	0.135		

BCG, Bacillus Calmette-Guérin; RC, radical cystectomy; BMI, body mass index; HBP, high blood pressure; DM, diabetes mellitus; PNI, prognostic nutrition index; CONUT, controlling nutritional status; AAPR, albumin-to-alkaline phosphatase ratio; NLR, neutrophil-to-lymphocyte ratio; PLR, platelet-to-lymphocyte ratio; LMR, lymphocyte ratio; SII, systemic immunoinflammatory index; CI, confidence interval; PFS, progression-free survival.

### Establishment of predictive nomograms for OS and PFS

**Figure 2:**
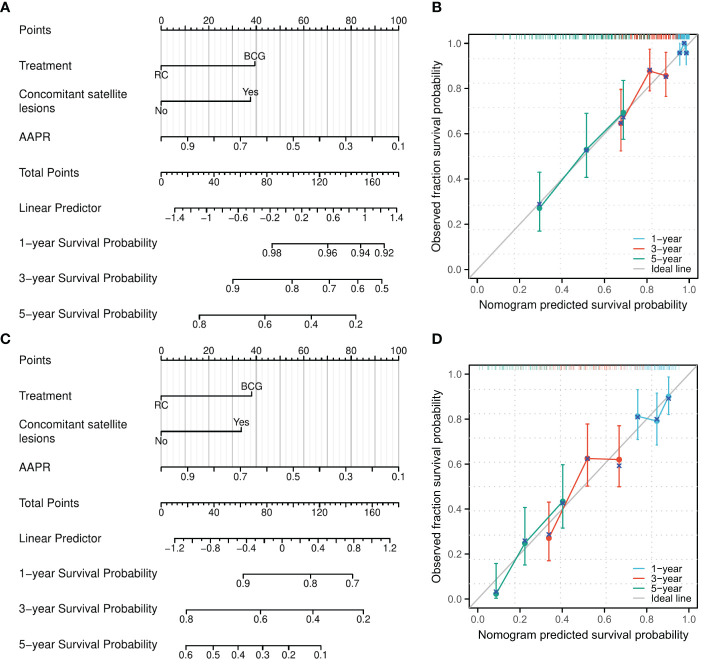
A nomogram model was established to predict the risk of OS and PFS for patients pathologically confirmed as high-grade T1 in initial and repeat transurethral resection of bladder tumor. **(A)** A nomogram for predicting 1-, 3-, and 5-year OS for patients pathologically confirmed as high-grade T1 in initial and repeat transurethral resection of bladder tumor. **(B)** Calibration of the nomogram model for OS. **(C)** A nomogram for predicting 1-, 3-, and 5-year PFS for patients pathologically confirmed as high-grade T1 in initial and repeat transurethral resection of bladder tumor. **(D)** Calibration of the nomogram model for PFS. AAPR, albumin-to-alkaline phosphatase ratio.

According to the results of multivariate logistic analysis, nomograms predicting OS [C-index: 0.665, 95% confidence interval (CI): 0.629–0.700] and PFS (C-index: 0.593, 95% CI: 0.564–0.622) were obtained (**Figures 2A, C**). Calibration curves indicated that the nomograms had a good degree of fit (**Figures 2B, D**).

## Discussion

Bladder cancer not only has a high prevalence rate but also imposes a higher lifetime treatment cost than all other forms of cancer (1, 4, 15). Therefore, determining the most effective treatment measures for patients with different clinicopathological stages and grades to improve PFS and OS has significant clinical implications. There exists the possibility of inadequate tumor grading and staging, as well as residual tumors after the initial TURBT surgery, which are significant factors leading to a poor prognosis of NMIBC. Although the benefits of re-TURBT for patients remain controversial, it proves beneficial particularly for those patients where the detrusor muscle was not obtained during the initial TURBT surgery (16). For these reasons, authorities such as the EAU and AUA recommend that patients with NMIBC who meet the criteria for secondary TURBT should undergo re-TURBT 2 to 6 weeks after the initial TURBT (4, 17). Nevertheless, the choice of treatment for patients at high risk with NMIBC with a high-grade T1 pathological result, whether BCG instillation or RC treatment, remains a point of contention in contemporary clinical practices (5–7). Our study conducted a long-term follow-up and compared the differences in OS and PFS among patients who underwent BCG instillation or RC treatment. We observed a significant difference in OS and PFS beginning from three years post-treatment, suggesting a long-term superiority of RC over BCG instillation. Hence, our study contributes valuable insights for the strategic selection of treatments for this patient cohort.

It should be noted that the prognosis varies among different histologies of bladder tumors (18). For patients with urothelial carcinomas, clear-cell, plasmacytoid, small-cell, and sarcomatoid variant histologies (VH) have poorer disease-specific survival, while those with lymphoepithelioma-like VH tend to have better outcomes. Suh et al. (19) conducted a study on 45 patients with high-risk NMIBC with squamous or glandular histological variants, of whom 30 underwent BCG instillation and 15 underwent RC. The 5-year OS (p=0.893) and PFS (p=0.811) rates were similar. The findings from the study by Lonati et al. (6) on 188 patients with high-risk NMIBC echoed these results. A large-scale multi-center study from Sweden followed up 3862 patients with high-risk NMIBC who received BCG instillation treatment and 687 who received RC treatment and found that the 5-year PFS in the BCG group was superior to that in the RC group (87% vs. 71%) (5). Hautmann et al. (20), in their study of 1,100 patients with malignant bladder tumors, posited that patients with high-risk NMIBC could benefit from immediate RC surgery, with a 5-year OS exceeding 80%. Our research data indicated that for patients with high-risk NMIBC with high-grade T1 staging, there were no statistically significant differences in OS and PFS within the first 2 years, regardless of whether they received BCG or RC treatment. However, starting in the third year, patients receiving RC demonstrated a significant survival advantage in terms of both OS and PFS. Although BCG instillation therapy demonstrated similar clinical benefits to RC treatment in the initial two years, its effectiveness diminished over time. Therefore, for this group of patients, assuming conditions permit, opting for RC treatment over BCG instillation therapy would yield better clinical outcomes.

The presence of satellite lesions can increase the probability of residual tumors after surgery, thereby increasing the patient’s chance of tumor recurrence (4, 17). This can ultimately delay treatment and negatively affect patient survival. Our study confirmed that the presence of satellite lesions was an independent risk factor for OS and PFS. AAPR, the ratio of albumin to alkaline phosphatase, was first introduced by Anthony W H Chan et al. (21) in 2015 as an independent risk factor for PFS after liver cancer surgery. Researches conducted by Shijie Li et al. (10) and Zhao et al. (22) found that AAPR is an independent risk factor for prognosis after surgery for malignant bladder tumors. This conclusion aligns with our study showing that AAPR is an independent risk factor for OS and PFS in patients with high-risk NMIBC classified as a high-grade T1 stage. A retrospective study involving 516 patients diagnosed with bladder cancer revealed that PNI served as an independent predictor for OS and PFS (8). This discovery highlights the potential for PNI to be a reliable, novel biomarker for bladder cancer. Concurrently, another retrospective study focused on delineating the relationship between the CONUT score and bladder cancer (9). The study found that a higher CONUT score predicted worse recurrence-free survival, OS, and cancer-specific survival. Although our study identified a correlation between PNI and the prognosis of high-risk, high-grade T1 NMIBC patients, PNI was not found to be an independent risk factor for postoperative OS or PFS in this patient population. Neutrophils are important inflammatory cells in humans. Previous studies have found that under the influence of the tumor microenvironment, they promote tumor cell proliferation and angiogenesis, ultimately accelerating tumor development (11). Lymphocytes are considered the primary executors of immune functions. However, owing to the high metabolic nutritional consumption of tumor tissues within the body, patients may experience a relative reduction in lymphocytes owing to progressively declining immune function. The primary role of platelets is to promote coagulation, but they also contain a large number of angiogenesis regulatory proteins that can be utilized by tumor cells and tumor matrix cells to promote the formation of new blood vessels, thereby accelerating tumor cell proliferation and invasion (11). The inflammatory indices derived from these indicators were related to the prognosis of bladder tumors (11, 23). However, we did not find these indices to be independent risk factors for the prognosis of high-risk high-grade T1 NMIBC.

Our study has certain limitations. Initially, this was a single-center study with a relatively small sample size; hence, the conclusions drawn require further validation through subsequent multi-center studies involving larger sample sizes. Second, our study exclusively targeted patients with high-risk NMIBC classified as a high-grade T1 stage. We aimed to extend our research to explore the OS and PFS outcomes in all types of patients with high-risk NMIBC. Thirdly, our analysis fell short as it omitted T1 substaging, an evolving and pivotal prognostic classification. This oversight could potentially impact the scope of our study and depth of insight into the disease prognosis. Going forward, we are intent on exploring the prognostic disparities between T1 substaging patients administered BCG instillation therapy versus those subjected to RC (24). Finally, the duration of follow-up in our study may be considered too short; however, we plan to continue these follow-ups in the future.

In summary, our study found that for patients with high-risk NMIBC classified as high-grade T1 stage, RC could confer survival benefits compared with BCG instillation if satellite lesions, and low AAPR are concurrently present. 

## Data availability statement

The datasets presented in this study can be found in online repositories. The names of the repository/repositories and accession number(s) can be found below: All original raw data of this study can be accessed from https://figshare.com/s/9c42afea3f51c6f1159e.

## Ethics statement

The studies involving humans were approved by the ethics committee of the First Affiliated Hospital of Soochow University. The studies were conducted in accordance with the local legislation and institutional requirements. The participants provided their written informed consent to participate in this study.

## Author contributions

SZ: Writing – original draft, Visualization, Software, Resources, Investigation, Formal analysis, Data curation. CH: Writing – original draft, Software, Resources, Formal analysis, Data curation. YY: Writing – original draft, Visualization, Validation, Investigation, Formal Analysis, Data curation. LY: Writing – review & editing, Supervision, Project administration, Methodology, Funding acquisition. OJ: Writing – review & editing, Supervision, Project administration, Methodology. ZZ: Writing – review & editing, Supervision, Software, Project administration, Methodology, Conceptualization.
